# Autoimmune hepatitis after COVID-19 vaccination

**DOI:** 10.3389/fimmu.2022.1035073

**Published:** 2022-11-25

**Authors:** Han Zheng, Ting Zhang, Yiyao Xu, Xin Lu, Xinting Sang

**Affiliations:** Department of Liver Surgery, Peking Union Medical College Hospital, Chinese Academy of Medical Sciences & Peking Union Medical College (CAMS & PUMC), Beijing, China

**Keywords:** COVID-19, autoimmune hepatitis (AIH), vaccination, SARS-CoV-2, liver

## Abstract

Vaccination is one of the most vigorous ways to intervene in the severe acute respiratory syndrome coronavirus 2 (SARS-CoV-2) pandemic. Cases of autoimmune hepatitis (AIH) after coronavirus disease (COVID-19) vaccination have been increasingly reported. Twenty-seven cases of AIH are summarized in this study, providing emerging evidence of autoimmune reactions in response to various COVID-19 vaccines, including in patients with special disease backgrounds such as primary sclerosing cholangitis (PSC), liver transplantation, and previous hepatitis C virus (HCV) treatment. Molecular mimicry, adjuvants, epitope spreading, bystander activation, X chromosome, and sceptical hepatotropism of SARS-CoV-2 may account for, to some extent, such autoimmune phenomena. Immunosuppressive corticosteroids perform well with or without azathioprine in such post-COVID-19-vaccination AIH. However, determination of the exact mechanism and establishment of causality require further confirmation.

## Introduction

The 2019 coronavirus disease (COVID-19) pandemic, caused by the novel Severe Acute Respiratory Syndrome Coronavirus-2 (SARS-CoV-2), has resulted in enormous morbidity and mortality rates globally. Up to August 2022, more than 586 million confirmed cases and more than 6 million deaths have been reported worldwide, with close to 12 billion total vaccine doses administered (1). One of the most effective strategies for mitigating COVID-19 is vaccination; this can create an immune barrier in the general population, attenuating the speed and scope of SARS-CoV-2 transmission. Vaccines were developed at an unprecedented speed and scale to combat this global crisis common to all human beings (2). The platforms used in COVID-19 vaccines include classical and novel, such as viral vector (replicating and non-replicating), nucleic acid (RNA and DNA), protein-based (protein subunit and virus-like particle), and whole virus (inactivated or weakened) (3). The two most common vaccine platforms currently used are mRNA-based (Pfizer-BioNTech and Moderna) and adenovirus vector-based (ChAdOx1 nCoV-19) vaccines (4). Multiple rare adverse events have occurred after COVID-19 vaccination, including myocarditis (5), vaccine-induced immune thrombotic thrombocytopenia (VITT) (6, 7), IgA vasculitis (8), and autoimmune diseases. Autoimmune hepatitis (AIH) is a type of autoimmune liver disease. Autoimmune liver diseases are a group of immune-disordered conditions, including AIH, primary sclerosing cholangitis (PSC), and primary biliary cholangitis (PBC) (9). Recently, a good number of autoimmune hepatitis cases following COVID-19 vaccination using different technological platforms have been reported, with variable characteristics. Given the emerging link between certain COVID-19 vaccines and autoimmune diseases, the risk of inducing autoimmune diseases by immunization has sparked public concern. Previous studies have demonstrated that SARS-CoV-2 infection could trigger autoimmunity (10), and that vaccines, as a general conception, could initiate regular or irregular autoimmune reactions in organisms. However, association between a specific type of COVID-19 vaccine and liver autoimmune phenomena remains nebulous, for which the exact mechanism is controversial; an agreement has not yet been reached. In this study, plausible mechanisms for immune activation triggered by COVID-19 vaccination are discussed and postulated. The purpose of this review is to summarize a set of cases on autoimmune hepatitis occurring after COVID-19 vaccination; and to unravel potential underlining mechanisms regarding the relationships between these autoimmune phenomena and COVID-19 vaccines.

## AIH after COVID-19 vaccination

The first case of AIH after COVID-19 vaccine was reported by Bril et al. (11), who described a 35-year-old female in her third month postpartum. This Caucasian woman developed pruritus, choluria, and jaundice 1 week after the first dose of the Pfizer-BioNTech COVID-19 vaccine. Antinuclear antibody (ANA) and double-stranded DNA antibodies were positive, while immunoglobulin G (IgG) levels were not increased, as is typical for AIH. A liver biopsy showed pan-lobular hepatitis with intense lymphoplasmacytic infiltration and rosette formation. An atypical feature observed was the presence of eosinophils, which are more commonly seen in liver injuries caused by drugs or toxins. The Revised Original Score for autoimmune hepatitis pretreatment was 18 (a score > 15 suggests definite AIH). However, her recent pregnancy was considered to be a confounding factor that interfered with the immune system (12). Quickly after this first case, Rocco et al. reported another case: an 80-year-old woman with previous Hashimoto’s thyroiditis who developed AIH a week after her second dose of the Pfizer-BioNTech COVID-19 vaccine. Her manifestations were jaundice, hyperchromic urine, positive ANA, interface hepatitis, and significantly elevated liver enzymes and IgG levels (13). In addition, a 41-year-old female had received the SARS-CoV-2 Moderna vaccine (mRNA-1273) prior to the presentation of AIH, presenting a positive ANA and anti-smooth muscle antibody (ASMA) as well as typical histological findings (14). Specially, anti-soluble liver antigen (anti-SLA) was positive in this patient, which prompted the possibility that homology between the SARS-CoV-2 spike protein and the soluble liver antigen was present. Another typical AIH case after receiving the Moderna vaccine (mRNA-1273) was reported in a 56-year-old woman without previous morbidity, who presented greatly increased levels of liver enzymes, IgG, and autoimmune antibodies (15). Moreover, eosinophils were also identified in her liver biopsy. Furthermore, three cases were reported after a different kind of vaccine called ChAdOx1 nCoV-19 vaccine (Oxford-AstraZeneca) was administered. The first case was a 36-year-old male who presented only mild febrile as a symptom; his Revised Original Score for AIH pretreatment was 15 (a score of 10–15 suggests probable AIH) (16). The second case was a 38-year-old woman with previous hypothyroidism, who developed jaundice, fever, malaise and choluria, as well as a positive ANA (17). The third case was another male patient whose liver biopsy results showed typical traits: lymphoplasmacytic infiltration, rosette formation, and emperipolesis (a phenomenon where lymphocytes penetrate hepatocytes), but with atypically negative ANA or ASMA (17). Similar to this case, a 43-year-old woman developed jaundice and pruritis after receiving the COVID-19 vaccine; both ANA and ASMA were negative while a liver biopsy showed interface hepatitis with lymphocytic infiltration (18). Afterwards, a series of AIH cases following the Moderna COVID-19 vaccination were reported. A 71-year-old female, reported by McShane et al. (19), a 63-year-old male, reported by Gheilmetti et al. (20), and a 59-year-old female, reported by Shroff et al. (21) developed AIH, with compatible histological findings and positive ANA or ASMA. A 76-year-old woman with a special medical history of prior COVID-19 infection developed choluria, malaise, markedly elevated IgG levels, and positive ANA and ASMA two days after receiving her first vaccine dose (22). A 36-year-old woman with previous history of the autoimmune condition PSC, was asymptomatic, with typical AIH histological findings and positive ANA (23). Moreover, three cases; a 47-year-old male (24), a 65-year-old female (25), and a 52-year-old female (26) developed AIH, with typical histological findings after receiving the Moderna vaccine. In addition, after receiving the Pfizer/BioNTech (BNT162b2) vaccination, a 61-year-old woman (27) developed jaundice and anorexia, with a Simplified Diagnostic Score of 7 (results ≥ 7 suggest definite AIH), while another 40-year-old woman (28) presented no symptoms, but significantly elevated serum transaminases. Erard et al. reported three patients with AIH caused by different vaccines: Pfizer/BioNTech, Moderna, and ChAdOx1 nCoV-19. The three cases exhibited similar symptoms; jaundice, pruritis, malaise, and similar histological features: diffuse hepatitis with lobular and portal lymphoplasmacytic penetration, interface hepatitis, and hepatocyte necrosis (29). A 79-year-old male who was the oldest male case thus far, despite clinical manifestations of jaundice, pruritus, and choluria after his first dose of the ChAdOx1 nCoV-19 vaccination, received the second dose and was then diagnosed to present typical features of AIH (30). Another case with PSC as an autoimmune background was a 63-year-old female, who developed jaundice and pruritis after ChAdOx1 nCoV-19 vaccine administration; however, her biopsy results and immunity investigation were not very typical (31). Kang et al. reported that a young, 27-year-old woman who developed jaundice, fever, choluria, and malaise a week after her second dose of the Pfizer/BioNTech vaccine, was diagnosed as definite AIH (32). An interesting case of a 32-year-old female, who underwent a liver transplantation for AIH in 2014 and was maintained in immunosuppression status, had an unexpected ALT elevation 28 days after her third dose of the Pfizer/BioNTech vaccine; her biopsy results showed significant lymphoplasmacytic inflammation and interface hepatitis, with focal rosette and emperipolesis (33). Hasegawa et al. (34) reported an 82-year-old woman who had a previous HCV infection and then tested negative for HCV RNA. Thus far, she was the oldest female case diagnosed as AIH positive after COVID-19 vaccination. The first case of AIH from an inactivated vaccine was a 52-year-old female who developed jaundice and malaise after her second dose of the CoronaVac vaccine (Sinovac Biotech, China). Her liver biopsy showed interface hepatitis with lymphoplasmacytic infiltration (35).

**Table 1** shows current case reports published in the recent literature, as described above. A total of 27 cases (11, 13–35) were described, with ages ranging from 27 to 82, including 22 females and 5 males. The trigger vaccine was the Moderna vaccine (mRNA-1273) in 11 cases, the Pfizer/BioNTech vaccine (BNT162b2) in 9 cases, the ChAdOx1 nCoV-19 vaccine (AZD1222) in 6 cases, and the CoronaVac vaccine (Sinovac Biotech, China) in 1 case. The first, second (or both), and even the third dose of vaccines were discovered to have a relationship with AIH. The latency time from vaccination to the onset of symptoms ranged from 1 day to approximately 1 month. Almost all subjects developed jaundice in the process, as well as other symptoms such as pruritis, fever, malaise, and anorexia. All cases did not use any hepatotoxic drug/agent or herbal remedies and did not consume or consumed a minimal of alcohol. Interferential infections such as hepatitis A virus (HAV), hepatitis B virus (HBV), hepatitis C virus (HCV), etc., were excluded in almost all cases.

**Table 1:** Autoimmune hepatitis following COVID-19 vaccination.

No	Age	Sex	Pre-existing morbidities	Concurrent medications and alcohol consumption	Vaccine type	Dose	Time of onset (days)	Symptoms	Histological features	AIH score	TB(μmol/L)	ALT(U/L)	AST(U/L)	ALP(U/L)	GGT(U/L)	IgG(g/L)	ANA	ASMA	Treatment	First author, year
1	35	F	Postpartum, gestational hypertension	Labetalol	Pfizer/BioNTech (BNT162b2)	1st	7	Jaundice, pruritis, choluria	Interface hepatitis, lymphoplasmacytic infiltration, rosettes, eosinophils	Revised Original Score pretreatment=18 (definite)	82.08	2001	754	170	–	10.81	1:1280	(-)	Prednisone	Bril, 2021 (11)
2	80	F	Hashimoto’s thyroiditis, glomerulonephritis	Levothyroxine, pravastatin, aspirin	Pfizer/BioNTech (BNT162b2)	2nd	7	Jaundice, choluria	Interface hepatitis, lymphoplasmacytic infiltration	Revised Original Score pretreatment=19 (definite)	179.55	1186	1401	243	524	35	1:160	(-)	Prednisone	Rocco, 2021 (13)
3	41	F	Premature ovarian failure	Substitutive hormonal therapy	Moderna (mRNA-1273)	Both 1st and 2nd	7	Abdominal pain, jaundice, choluria, anorexia	Interface hepatitis, lymphoplasmacytic infiltration	Simplified diagnostic score=8 (definite)	39.33	1312	993	190	209	20.8	1:80	1:40	Prednisone	Londoño, 2021 (14)
4	56	F	None	Rosuvastatin	Moderna (mRNA-1273)	1st	1	Jaundice, malaise, anorexia	Interface hepatitis, lymphoplasmacytic infiltration, rosettes, eosinophils	Revised Original Score pretreatment=16 (definite)	102	1701	1124	298	–	32.6	(+)	(+)	Budesonide	Tan, 2021 (15)
5	36	M	Hypertension	Olmesartan	ChAdOx1 nCoV-19 (AZD1222)	1st	26	Mild fever	Interface hepatitis, lymphocytic infiltration	Revised Original Score pretreatment=15 (probable)	17	1774	633	118	136	12.8	1:160	(-)	Prednisolone	Clayton-Chubb, 2021 (16)
6	38	F	Hypothyroidism	Levothyroxine	ChAdOx1 nCoV-19 (AZD1222)	1st	20	Jaundice, fever, malaise, choluria	Lymphoplasmacytic infiltration, eosinophils	Revised Original Score pretreatment=15 (probable)	48.906	1025	1101	–	–	16.5	1:80	(-)	Prednisolone	Rela, 2021 (17)
7	62	M	Diabetes mellitus	–	ChAdOx1 nCoV-19 (AZD1222)	1st	16	Jaundice, fever, anorexia	Lymphoplasmacytic infiltration, rosettes, Emperipolesis, eosinophils	Probable diagnosis	328.32	1094	1361	–	–	–	(-)	(-)	Prednisolone	Rela, 2021 (17)
8	43	F	Dyslipidemia, venous insufficiency	Ginkgo-biloba	Pfizer/BioNTech (BNT162b2)	Both 1st and 2nd	15	Jaundice, pruritis	Interface hepatitis, lymphocytic infiltration	–	299.934	52	51	–	–	–	(-)	(-)	Steroids	Lodato, 2021 (18)
9	71	F	Osteoarthritis	–	Moderna (mRNA-1273)	1st	4	Jaundice	Interface hepatitis, lymphoplasmacytic infiltration, eosinophils	–	270	1067	–	217	–	21.77	–	1:2560	Prednisolone	McShane, 2021 (19)
10	63	M	Type 2 diabetes, ischemic heart disease	Metformin, acetylsalicylic acid, rosuvastatin, 10g/d alcohol	Moderna (mRNA-1273)	1st	7	Jaundice, malaise, anorexia	Interface hepatitis, lymphoplasmacytic infiltration, eosinophils	–	204.8	1038	1127	192	536	19.96	1:640	(-)	Prednisone	Gheilmetti, 2021 (20)
11	59	F	None	Tylenol	Moderna (mRNA-1273)	1st	31	No mention	Severe inflammation, lymphocytic infiltration	Simplified diagnostic score=6 (probable)	251.37	869	–	367	–	17.50	1:640	–	VI steroids	Shroff, 2021 (21)
12	76	F	Hashimoto’s thyroiditis, prior COVID-19 infection, urothelial carcinoma	Levothyroxine, midodrine, zolpidem	Moderna (mRNA-1273)	1st	2	Choluria, malaise	Interface hepatitis, lymphoplasmacytic infiltration, rosettes	Simplified diagnostic score =8 (definite)	65	579	811	124	361	39.4	1:1280	1:1280	Prednisolone, azathioprine	Vuille-Lessard, 2021 (22)
13	36	F	Primary sclerosing cholangitis, ulcerative colitis	–	Moderna (mRNA-1273)	1st	11	None	Interface hepatitis, lymphoplasmacytic infiltration, rosettes, eosinophils	–	23.94	588	581	–	–	24.75	1:2560	(-)	Prednisone	Zhou, 2021 (23)
14	47	M	None	Minimal alcohol	Moderna (mRNA-1273)	Both 1st and 2nd	3	Jaundice, malaise	Interface hepatitis, lymphoplasmacytic infiltration, emperipolesis, eosinophils	–	190	1048	–	229	–	25.1	(+)	–	Prednisolone	Tun, 2021 (24)
15	65	F	JAK2 V617F-positive polycythemia vera	Acetylsalicylic acid, sertraline, esomeprazole, pegylated interferon	Moderna (mRNA-1273)	1st	14	Abdominal pain, jaundice, choluria	Interface hepatitis, lymphoplasmacytic infiltration	Simplified diagnostic score =8 (definite)	19.494	1092	1056	24	329	20	1:100	(-)	Prednisolone	Garrido, 2021 (25)
16	52	F	None	–	Moderna (mRNA-1273)	1st	14	Jaundice, malaise	Interface hepatitis, lymphoplasmacytic infiltration, rosettes, eosinophils	Simplified diagnostic score (definite)	154.926	936	350	169	810	23.96	1:320	(+)	Prednisolone, azathioprine	Goulas, 2021 (26)
17	61	F	Hashimoto’s thyroiditis, hypertension	Valsartan, levothyroxine	Pfizer/BioNTech (BNT162b2)	No mention	30	Jaundice, malaise, anorexia	Interface hepatitis, lymphocytic infiltration	Simplified diagnostic score =7 (definite)	201.78	455	913	436	292	42.6	1:100	1:100	Prednisolone, azathioprine	Avci, 2021 (27)
18	40	F	Sarcoidosis	–	Pfizer/BioNTech (BNT162b2)	2nd	30	None	Interface hepatitis, lymphoplasmacytic infiltration	–	–	–	4×ULN	–	–	24	1:640	(-)	Prednisolone	Palla, 2021 (28)
19	80	F	No history of autoimmune disease	–	Pfizer/BioNTech (BNT162b2)	2nd	10	Jaundice, pruritis, malaise	Interface hepatitis, lymphoplasmacytic infiltration	–	78	541	583	–	–	20.9	(+)	(-)	Steroids	Erard, 2021 (29)
20	73	F	No history of autoimmune disease	–	Moderna (mRNA-1273)	1st	21	Jaundice, pruritis, malaise	Interface hepatitis, lymphoplasmacytic infiltration	–	334	1027	1163	–	–	18	(+)	(-)	Steroids	Erard, 2021 (29)
21	68	F	No history of autoimmune disease	–	ChAdOx1 nCoV-19 (AZD1222)	1st	20	Jaundice, pruritis, malaise	Interface hepatitis, lymphoplasmacytic infiltration	–	752	2029	2314	–	–	18.5	(+)	(-)	Steroids	Erard, 2021 (29)
22	79	M	None	–	ChAdOx1 nCoV-19 (AZD1222)	Both 1st and 2nd	15	Abdominal pain, jaundice, pruritis, choluria, anorexia	Interface hepatitis, lymphocytic infiltration, eosinophils	type I AIH (the IAIHG criteria)	203.49	1994	2003	–	–	20.58	1:80	(+)	Prednisone, azathioprine	Camacho-Domínguez, 2022 (30)
23	63	F	Ulcerative colitis, primary sclerosing cholangitis	Ursodeoxycholic acid, mesalazine, azathioprine, atorvastatin	ChAdOx1 nCoV-19 (AZD1222)	1st	14	Jaundice, pruritis	Interface hepatitis	–	313	354	505	299	65	20.3	(+)	–	Prednisolone	Affendi, 2022 (31)
24	27	F	–	–	Pfizer/BioNTech (BNT162b2)	2nd	7	Jaundice, fever, choluria, anorexia	Interface hepatitis, lymphoplasmacytic infiltration, rosettes, eosinophils	Revised Original Score pretreatment=18 (definite)	147.06	1478	1004	182	–	16.41	1:80	(-)	Prednisolone	Kang, 2022 (32)
25	32	F	Liver transplantation for AIH, hypertension	Tacrolimus, azathioprine, prednisolone, aspirin, vitamin D, atenolol	Pfizer/BioNTech (BNT162b2)	3rd	28	None	Interface hepatitis, lymphoplasmacytic infiltration, rosettes, emperipolesis	–	29	288	–	115	–	–	–	–	Methylprednisolone, prednisolone	Mahalingham, 2022 (33)
26	82	F	HCV treatment	Ledipasvir, sofosbuvir	Pfizer/BioNTech (BNT162b2)	1st	7	Malaise, anorexia	Interface hepatitis, lymphoplasmacytic infiltration	Revised Original Score pretreatment	377.91	1097	1682	77	104	1809	(+)	–	Prednisolone	Hasegawa, 2022 (34)
27	52	F	Hypertension, dyslipidemia	Simvastatin; amlodipine; losartan	CoronaVac (Sinovac Biotech, China)	2nd	7	Jaundice, malaise	Interface hepatitis, lymphoplasmacytic infiltration	AIH cirrhosis	155.61	180	566	228	–	27.12	1:160	1:400	Prednisolone, azathioprine	Mekritthikrai, 2022 (35)

AIH, autoimmune hepatitis; TB, total bilirubin; ALT, alanine aminotransferase; AST, aspartate aminotransferase; ALP, alkaline phosphatase; GGT, gamma glutamyl transferase; ANA, antinuclear antibody; ASMA, anti-smooth muscle antibody.

In terms of the biochemistry results, the total bilirubin (TB) recorded in 26 of the 27 cases ranged from 17 mol/L to 752 mol/L. Alanine aminotransferase (ALT) recorded in 26 of the 27 cases ranged from 52 U/L to 2029 U/L. Aspartate aminotransferase (AST) recorded in 23 of the 27 cases varied from 51 U/L to 2314 U/L. Alkaline phosphatase (ALP) recorded in 19 of the 27 cases ranged from 24 U/L to 436 U/L. Gamma glutamyl transferase (GGT) recorded in 11 of the 27 cases ranged from 65 U/L to 810 U/L. These results suggest that normal liver biochemistry tests could not serve to exclude a possible diagnosis of AIH. Moreover, these liver functional indexes may be extremely high in a patient with AIH.

Scoring parameters [Simplified Diagnostic Criteria or Revised Original Diagnostic Criteria for AIH (36–38)] were used to define the reliabilities of liver injuries in 12 cases (6 Simplified, 6 Revised), resulting in nine cases with definite AIH and three cases with probable AIH. All patients were treated with steroid therapy (mostly prednisone or prednisolone), with or without azathioprine. All patients turned out well, except two: one died after 30mg/d prednisolone treatment, followed by five cycles of therapeutic plasma exchange and was unable to undergo a liver transplantation due to socio-economic constrains (17); another had a poor course, developing hepatic encephalopathy, liver failure, and sepsis, and died 3 days after the liver failure (29).

## Brief overview of COVID-19 vaccines and AIH

Broadly used vaccines, such as the Moderna (mRNA-1273), Pfizer/BioNTech (BNT162b2), and ChAdOx1 nCoV-19 (AZD1222) vaccines can be classified into mRNA-based (Moderna and Pfizer-BioNTech) and adenovirus vector-based (AZD1222) vaccines. The protective mechanism of mRNA-based vaccines against SARS-CoV-2 is as follows: the delivering mRNA, which encodes the SARS-CoV-2 spike protein, is taken up by host cells and decoded by ribosomes to release the spike proteins; these manufactured spike proteins are then recognized and presented by the host’s underlying immune pathways to provoke a robust CD8+ and CD4+ T cell-mediated response (39). In order to prolong its biological half-life and enhance its stimulating capability, the mRNA is enveloped in lipid nanoparticles (LNP) (40). Different from mRNA-based vaccines, adenovirus vector-based vaccines use replication-deficient adenoviruses as safe platforms to carry SARS-CoV-2 genes encoded with spike proteins, which then enter the host’s nucleus and are transcribed into the corresponding mRNA, and finally, spike proteins are translated (40, 41). Adenovirus vector-based vaccines, by mimicking a natural viral infection, comprehensively induce effective humoral and cellular immune responses against SARS-CoV-2 (41). The CoronaVac vaccine (Sinovac Biotech, China), as an inactivated vaccine, provides protection by stimulating the immune system with a killer pathogen, which loses its replication and invasion properties, but retains its antigen-specificity in combination with adjuvants (42).

The liver is a vital frontline immune organ (43). Individual liver functional units, named lobules, hide in a heterogeneous, complicated, and elaborate system (44). The default immune status of the liver is anti-inflammatory or immunotolerant; however, under appropriate conditions, the liver is able to provide a rapid and robust immune response (43). The basis of AIH pathogenesis is the interaction between a susceptible host and precipitating factors from environment (45). Through molecular mimicry, predisposing factors may impair immunoregulatory homeostasis *via* a comprehensive network of cells including CD4+T cells, Treg cells, CD8+T cells, and autoantibodies produced by B cells (46, 47). Similar to other autoimmune diseases, AIH is associated with non-organ-specific antibodies in the context of hepatic autoimmunity (48).

## Possible underlining mechanism

### Molecular mimicry and immune crossreaction

Molecular mimicry is defined as the significant homology between microbial agents and the human host, while immune crossreaction refers to the crossreactivity of the immune system in targeting pathogenic antigens and destroying similar human proteins at the same time (49). As for pathogens of SARS-CoV-2, it had been illustrated that its spike proteins shared 13 of 24 pentapeptides with human lung surfactant proteins, whereby immune crossreaction occurred and SARS-CoV-2 first invaded the respiratory system (10, 50). One study, in which a mouse monoclonal antibody against the recombinant SARS coronavirus spike protein was applied to SARS-CoV-2 proteins as well as to 50 different tissue antigens, demonstrated that 21 out of 50 tissue antigens exhibited moderate to strong reactions with SARS-CoV-2 antibodies (51). The series of tissue antigens presenting the strongest reactions included transglutaminase 3 (tTG3), transglutaminase 2 (tTG2), anti-extractable nuclear antigen (ENA), myelin basic protein (MBP), mitochondria, nuclear antigen (NA), α-myosin, thyroid peroxidase (TPO), collagen, claudin 5 + 6, and S100B (51). Concrete molecules that may be associated with the liver were mitochondrial and nuclear antigens. Antimitochondrial antibodies (AMA) were directed against antigenic components of the cellular mitochondria in different human organs and tissues, which were considered the serum hallmarks of primary biliary cholangitis (PBC) (52). In the case of AIH occurring after mRNA-1273 SARS-CoV-2 vaccination, an indirect immunofluorescence pattern was observed on triple rodent tissue, compatible with anti-mitochondrial antibody (AMA), which, however, was not positively presented for PBC-specific tests (20). Nuclear antigens (NA) were detected in the mouse liver nonhistone protein fraction by using antibodies in whole liver cells (53). Antibodies, found in sera from patients with primary biliary cholangitis and autoimmune chronic active hepatitis, could detect a nuclear protein of 54 kD (54).

### Adjuvants

An adjuvant is a substance that is added to a vaccine to stimulate and strengthen the magnitude and durability of the immune response (55). The promoting effects of adjuvants are achieved by various mechanisms (1): imitation of evolution-conservative molecules (e.g., bacterial cell walls, LPS, and unmethylated CpG-DNA) (2, 56); binding to Toll-like receptors, which are key molecules expressed by innate immune cells for detection (3, 57); strengthening of the activities of dendritic cells, lymphocytes, and macrophages (58); and (4) activation of the intracellular NLPR3 inflammasome, which mediates caspase-1 activation and the secretion of the proinflammatory cytokines IL-1β/IL-18 in response to microbial infection and cellular damage (59). After investigating the role of adjuvants in the pathogenesis of immune-mediated diseases, Shoenfeld and Agmon-Levin (60) introduced a concept named “Autoimmune (Auto-inflammatory) Syndrome Induced by Adjuvants: ASIA”. When restricted to COVID-19 vaccines, the main components of adjuvants in mRNA-based vaccines are lipid nanoparticles (LNPs), which encapsulate the core mRNA. In mouse models, LNPs could trigger inflammatory responses with massive neutrophil infiltration, could activate diverse inflammatory pathways, and could produce various inflammatory cytokines and chemokines (61). In vaccines, the core mRNA itself even plays the role of adjuvant, owing to the intrinsic immunostimulatory properties of RNA, which can be recognized by intracellular receptors such as Toll-like receptors 3 and 7, resulting in downstream activation of proinflammatory cytokines (62).

### Epitope spreading and bystander activation

Epitope spreading is defined as the alteration of epitope specificity from the initial, focused, and dominant epitope to subdominant and cryptic epitopes on a particular protein (intramolecular spreading) or other proteins (intermolecular spreading) (63). Epitope spreading is considered to not only protect against pathogens but also to propagate autoimmunity (64). Bystander activation occurs when CD8+ T, CD4+ T, or B cells are activated in an antigen-independent manner (65), which is beneficial for pathogen clearance and may play a role in the development of AIH (66). Vadalà et al. (67) provided the viewpoint that microbial agents may release sequestered self-antigens from host tissues that activate antigen-presenting cells and dormant autoreactive T-helper cells. Epitope spreading and bystander activation processes may be even more prominent in genetically predisposed individuals (68). A hypothesis was proposed whereby COVID-19 vaccination triggered pre-existing underlying dysregulated pathways, rather than provoking *de novo* immune-mediated adverse events (69) in susceptible groups presenting an autoimmunity background such as PSC, Hashimoto’s thyroiditis, etc.

### X chromosome and female susceptibility

Despite similar COVID-19 infection rates in the two sexes, males are more vulnerable to morbidity and mortality (70). This has been explained by differences in hormonal profiles, chromosomal compositions, and gender behaviors. Females apparently produce higher numbers of antibodies while simultaneously exhibiting higher rates of side effects, indicating a stronger immune reactivity to a vaccine’s elements (70). The X chromosome carries several genes involved in the immune response, including Toll-like receptor 7 (TLR7), TLR8, and intereukin-1 receptor-associated kinase 1 (IRAK1) (71). TLR3, TLR7, and TLR9, which are female-biased, have been shown to provide protection against viruses by recognizing viral RNA and DNA (72). Due to the stimulatory effect of estrogens on the immune system, over-expression of X-linked genes provoke a stronger and more susceptible post-vaccination autoimmune reaction in females than males (73).

### Hepatotropism of SARS-CoV-2?

Although abnormal liver tests are common in SARS-CoV-2 infected patients, the direct impact of SARS-CoV-2 on the liver remains undetermined. Wang et al. proposed that the direct SARS-CoV-2 infection of liver cells significantly contributes to hepatic impairment; electron microscopy evidence identified typical spike structures in the cytoplasm of hepatocytes (74). However, these ultrastructure electron microscopy findings were considered to be normally occurring clathrin-coated vesicles inside hepatic cells, thus weakening the foundations of the direct-infection viewpoint (75). Human angiotensin-converting enzyme 2 (ACE-2) receptors, serving as entry points for the spike proteins in SARS-CoV-2 to recognize and bind to (76), were expressed on hepatocytes and more abundantly on some cholangiocytes, which implied that liver injury could be mediated *via* bile duct cells (77). Other studies (75, 77–80) suggested that the liver was only involved as a part of the severe systemic inflammatory disease, and abnormal liver tests in SARS-CoV-2 patients reflected the severity of the virus, rather than direct destruction by the virus.

## Discussion and conclusion

In light of the work presented above, a possible clue for COVID-19 vaccination–induced AIH can be extracted. The possible mechanisms that may occur behind the scenes include molecular mimicry, adjuvants, epitope spreading, bystander activation, X chromosome, and sceptical hepatotropism of SARS-CoV-2. For ordinarily developed AIH, initial treatments include high doses of immunosuppressive corticosteroids, which are then tapered gradually using azathioprine to minimize adverse effects (81). This fundamental treatment still works when extended to the more complex situation of AIH following COVID-19 vaccination.

An important question still remains however: should a second dose of a COVID-19 vaccine be administered when autoimmune hepatitis occurs following initial vaccination? Considering that the SARS-CoV2-antibody titration was weak two weeks after the first vaccine, a 36-year-old woman decided to get a second dose, under treatment with 50 mg prednisone. Without deterioration, gradual normalization of liver function indicators was detected (23). This case may serve as important evidence that a second dose of a COVID-19 vaccine can be administered with no major liver destruction occurring. However, a comparative study between the degree of vaccine-related risk and natural infection-related risk for the entire population or susceptible groups should be conducted for more credible support.

Eosinophils, which are often associated with drug-induced liver injury, were observed in the liver biopsies of eleven cases (82, 83). This raises the question whether COVID-19 vaccinations incite a phenomenon resembling drug-induced-AIH (DI-AIH) or trigger idiopathic AIH (84). The literature suggests a significant overlap between idiopathic AIH and DI-AIH cases.

According to previous studies, the distribution of age at AIH onset was thought to be bimodal, with peaks around puberty and between the 40 and 60 years of age (85). However, in a total of twenty-seven patients, seven subjects were older than 70, beyond the ordinary statistical age. Moreover, none of the AIH cases reported after COVID-19 vaccination were in adolescents. Therefore, further attention needs to be paid to the elderly and adolescent.

Indeed, evidence of COVID-19 vaccinations inducing AIH in this review was limited to case reports, case series, and letters to the editor. However, it may be presumed that, where there is smoke there is fire. The reliability of case reports fails to confirm whether the relationship between AIH and vaccines is casual or causal. The concurrent use of drugs (statins and antibiotics) that may trigger autoimmunity adds further confoundment and raises questions on the causality (84). Causality assessment requires further investigation by long-term follow-ups and future animal experiments. The exact mechanisms of autoimmune hepatitis induced by COVID-19 vaccinations are far from being completely elucidated.

It should be reinforced that such evidence should not be used as a tool to promote vaccine hesitancy in the general population. The advantages of vaccination against COVID-19, in terms of reduced severity and mortality, far outweigh the risks of AIH demonstrated in this review. As more and more vaccine doses are administered to a wide population, awareness should be raised regarding potential side effects. Current studies are restricted to the first and/or second doses of COVID-19 vaccinations. As third doses are globally administered, especially across Asia, data on the third dose need to be collected.

## Author contributions

HZ (First Author): Conceptualization, Data Curation, Formal Analysis, Writing-Original Draft TZ: Writing-Original Draft; YX (Corresponding Author): Supervision, Writing-Review and Editing; XL: Supervision, Writing-Review and Editing; XS: Supervision, Writing-Review and Editing. All authors contributed to the article and approved the submitted version.

## Funding

This study was funded by the National High Level Hospital Clinical Research Funding with funding number: 2022-PUMCH-C-049.

## Conflict of interest

The authors declare that the research was conducted in the absence of any commercial or financial relationships that could be construed as a potential conflict of interest.

## Publisher’s note

All claims expressed in this article are solely those of the authors and do not necessarily represent those of their affiliated organizations, or those of the publisher, the editors and the reviewers. Any product that may be evaluated in this article, or claim that may be made by its manufacturer, is not guaranteed or endorsed by the publisher.
